# Supplementary material for: Investigating the application value of the “Forward-Deployed Position” model in operating room support by the central sterile supply department
Source: PLoS One. 2026 May 21;21(5):e0348606. doi: 10.1371/journal.pone.0348606 (PMC13193391; doi:10.1371/journal.pone.0348606)
Supplement: S1 Data — This table presents the raw data underlying Table 1, showing the total number of cleanings, the number of re-cleanings, and the calculated re-cleaning rates for the control group (2023) and the observation group (2024). The control group had 71,850 cleanings with 756 re-cleanings (1.05%), while the observation group had 91,765 cleanings with 336 re-cleanings (0.37%). (DOCX) [file pone.0348606.s001.docx]

| Re-cleaning Rate | | | |
| --- | --- | --- | --- |
| Year | Number of Cleanings | Number of Re-cleanings | Re-cleaning Rate |
| 2023 | 71850 | 756 | 1.05% |
| 2024 | 91765 | 336 | 0.37% |
