# Supplementary material for: Investigating the application value of the “Forward-Deployed Position” model in operating room support by the central sterile supply department
Source: PLoS One. 2026 May 21;21(5):e0348606. doi: 10.1371/journal.pone.0348606 (PMC13193391; doi:10.1371/journal.pone.0348606)
Supplement: S2 Data — This table presents the raw data underlying Table 2, showing the total number of packages, the number passed, and the calculated packaging qualification rates for the control group (2023) and the observation group (2024). (DOCX) [file pone.0348606.s002.docx]

| Packaging qualification Rate | | | |
| --- | --- | --- | --- |
| Year | Number of Packages | Number Passed | Pass Rate (%) |
| 2023 | 71850 | 70452 | 98.05% |
| 2024 | 91765 | 90890 | 99.05% |
