# Supplementary material for: Investigating the application value of the “Forward-Deployed Position” model in operating room support by the central sterile supply department
Source: PLoS One. 2026 May 21;21(5):e0348606. doi: 10.1371/journal.pone.0348606 (PMC13193391; doi:10.1371/journal.pone.0348606)
Supplement: S4 Data — This table presents the raw data underlying Table 4, showing the total number of first scheduled surgeries, the number of on-time starts, and the number of delayed starts for the control group (2023) and the observation group (2024). (DOCX) [file pone.0348606.s004.docx]

| On-time Start Rate for the First Scheduled Surgery | | | |
| --- | --- | --- | --- |
| Year | Number of Surgeries | On-time Start | Delayed Start |
| 2023 | 7300 | 6251 | 1049 |
| 2024 | 7300 | 6939 | 361 |
