# Supplementary material for: Investigating the application value of the “Forward-Deployed Position” model in operating room support by the central sterile supply department
Source: PLoS One. 2026 May 21;21(5):e0348606. doi: 10.1371/journal.pone.0348606 (PMC13193391; doi:10.1371/journal.pone.0348606)
Supplement: Attachment — Submitted filename: Response to Reviewers.docx [file pone.0348606.s005.docx]

****Dear Editor and Reviewers,****

Thank you for your valuable comments and suggestions regarding our manuscript (ID: PONE-D-25-62281). We have carefully considered each point and have revised the manuscript accordingly. Below, we provide a point-by-point response to the comments.

****1. PLOS ONE style requirements****
We have reviewed the PLOS ONE style templates and confirmed that our manuscript meets the journal’s formatting requirements, including file naming conventions.

****2. Ethics statement****
We have moved the ethics statement to the Methods section, as requested. The statement no longer appears in any other section of the manuscript.

****3–5. Data availability and sharing restrictions****
We appreciate the journal’s emphasis on data transparency. In response to the comments regarding data sharing, we have now uploaded the de-identified dataset as Supporting Information files. Accordingly, we have updated the Data Availability Statement in the submission form to reflect that all underlying data are provided within the Supporting Information files.

We confirm that no ethical or legal restrictions prevent the sharing of this de-identified dataset, and therefore no exemption request is needed.

****6. Funding information****
We have corrected the inconsistency between the “Funding Information” and “Financial Disclosure” sections.

****7. LaTeX template****
We have updated our submission to use the PLOS LaTeX template, in accordance with the journal’s requirements.

****8.It is recommended that the manuscript be re-edited and the references updated.****

We have carefully revised the manuscript and updated all references to ensure they are current. The references now predominantly include articles published within the last five years. Additionally, we have standardized the citation format to comply with PLOS ONE style requirements, including the addition of missing DOIs, PMIDs, and PMCID numbers where applicable. We believe these revisions have improved the quality and timeliness of the literature cited.

We believe that these revisions have addressed all the concerns raised. We appreciate your time and effort in handling our manuscript and look forward to your further feedback.

Sincerely,
Mingjun Wu
515290614@qq.com
